# Efficacy of omega-3 fatty acids supplementation as an adjunct to nonsurgical periodontal therapy in periodontitis: a systematic review and meta-analysis of randomized controlled trials

**DOI:** 10.21142/2523-2754-1403-2026-303

**Published:** 2026-07-10

**Authors:** Anis Irmawati, Fiki Muhammad Ridho, Raed Labib, Ridwan Alfatah, Siska Maulidina Cahyani

**Affiliations:** 1 Department of Basic Dental Science and Public Health, Faculty of Dental Medicine, Universitas Airlangga. Surabaya, Indonesia. anis-m@fkg.unair.ac.id Department of Basic Dental Science and Public Health Faculty of Dental Medicine Universitas Airlangga Surabaya Indonesia anis-m@fkg.unair.ac.id; 2 Department of Dental Medicine, Faculty of Dental Medicine, Universitas Airlangga. Surabaya, Indonesia. fiki.muhammad.ridho-2020@fkg.unair.ac.id Department of Dental Medicine Faculty of Dental Medicine Universitas Airlangga Surabaya Indonesia fiki.muhammad.ridho-2020@fkg.unair.ac.id; 3 Department of Oral Surgery, Faculty of Dental Medicine, 21 September University of Medical and Applied Sciences. Sana’a, Yemen. raed.labib@21umas.edu.ye Department of Oral Surgery Faculty of Dental Medicine 21 September University of Medical and Applied Sciences Sana’a Yemen raed.labib@21umas.edu.ye; 4 Faculty of Medicine, Universitas Islam Negeri Walisongo. Semarang, Indonesia. ridwan.alfatah@walisongo.ac.id Faculty of Medicine Universitas Islam Negeri Walisongo Semarang Indonesia ridwan.alfatah@walisongo.ac.id; 5 Faculty of Dentistry, Universitas Brawijaya. Malang, Indonesia. siskamaulidinac@student.ub.ac.id Faculty of Dentistry Universitas Brawijaya Malang Indonesia siskamaulidinac@student.ub.ac.id

**Keywords:** omega-3 fatty acids, periodontitis, periodontal disease, dentistry, good health and well-being, host modulation therapy, ácidos grasos omega-3, periodontitis, enfermedad periodontal, odontología, buena salud y bienestar, terapia de modulación del huésped

## Abstract

**Introduction:**

This study aimed to evaluate the efficacy of omega-3 fatty acids (n3-FA) supplementation as adjunct to scaling and root planing (SRP) in patients with periodontitis.

**Methods:**

A systematic review of randomized controlled trials (RCTs) evaluating n3-FA supplementation as a single adjunct therapy for SRP was conducted through a comprehensive search in Scopus, PubMed, Web of Science, Cochrane Library, and EBSCO. The Cochrane RoB2 tool was used as a tool for evaluating risk of bias. The outcomes of probing pocket depth (PPD) and clinical attachment level (CAL) were analyzed using a random-effects model meta-analysis and reported as mean differences (MD) and 95% confidence intervals (CI). The certainty of evidence was evaluated using the GRADE approach. RevMan 5.4 software was used for all statistical analysis.

**Results:**

Nine RCTs were included in the meta-analysis. Compared with SRP alone, adjunctive n3-FA supplementation showed a greater reduction in PPD (MD = -0.66; 95% CI: -1.03, -0.30; p = 0.0003; I^2^ = 91%) and gain in CAL (MD = -0.69; 95% CI: -1.06, -0.32; p = 0.0002; I^2^ = 88%). Sensitivity and subgroup analyses yielded consistent effect directions, although the overall certainty of evidence was rated as low.

**Conclusion:**

Adjunctive n3-FA supplementation as a host modulation therapy may provide additional benefits when combined with SRP in patients with periodontitis. However, given the substantial heterogeneity and low level of evidence, these findings should be interpreted cautiously. Further high-quality, more rigorously designed RCTs are required to confirm their long-term efficacy and safety.

## RESUMEN

**Introducción:**

Este estudio tuvo como objetivo evaluar la eficacia de la suplementación con ácidos grasos omega-3 (AG-n3) como coadyuvante del raspado y alisado radicular (RAR) en pacientes con periodontitis.

**Métodos:**

Se realizó una revisión sistemática de ensayos controlados aleatorizados (ECA) que evaluaron la suplementación con AG n-3 como terapia coadyuvante única del RAR, mediante una búsqueda exhaustiva en Scopus, PubMed, Web of Science, Cochrane Library y EBSCO. La herramienta Cochrane RoB2 se utilizó para evaluar el riesgo de sesgo. Los desenlaces de profundidad de sondaje periodontal (PS) y nivel de inserción clínica (NIC) se analizaron mediante un metaanálisis de modelo de efectos aleatorios, y se informaron como diferencias de medias (DM) y sus intervalos de confianza (IC) del 95%. La certeza de la evidencia se evaluó utilizando el enfoque GRADE. El *software* RevMan 5.4 se utilizó para todos los análisis estadísticos.

**Resultados:**

Nueve ECA fueron incluidos en el metaanálisis. En comparación con el RAR solo, la suplementación coadyuvante con AG n-3 mostró una mayor reducción de la PS (DM = -0,66; IC del 95%: -1,03, -0,30; p = 0,0003; I^2^ = 91%) y una ganancia en el NIC (DM = -0,69; IC del 95%: -1,06, -0,32; p = 0,0002; I^2^ = 88%). Los análisis de sensibilidad y de subgrupos mostraron direcciones de efecto consistentes, aunque la certeza global de la evidencia se calificó como baja.

**Conclusión:**

La suplementación coadyuvante con AG n-3 como terapia de modulación del huésped puede proporcionar beneficios adicionales cuando se combina con el RAR en pacientes con periodontitis. Sin embargo, dada la heterogeneidad sustancial y el bajo nivel de evidencia, estos hallazgos deben interpretarse con cautela. Se requieren ECA adicionales de alta calidad y con diseños más rigurosos para confirmar su eficacia y seguridad a largo plazo.

## INTRODUCTION

Periodontitis represents a persistent inflammatory condition driven by an imbalance in the oral microbiome, which progressively damages the periodontium ^1^. The burden of this disease is high globally, as evidenced by a periodontitis prevalence of 62% between 2011 and 2020, with severe periodontitis accounting for 23.6% of cases ^(2, 3)^. Moreover, it is one of the primary contributors to tooth loss and has been linked to detrimental systemic diseases ^4^^-^^11^. Therefore, the implementation of effective, evidence-based strategies for managing periodontitis is essential to halt disease progression and reduce its impact on overall health.

Scaling and root planing (SRP), a nonsurgical periodontal therapy (NSPT), serves as the first-line mechanical approach in the management of periodontitis that aims to eliminate the disease-causing biofilm, thereby achieving adequate infection and inflammation control ^(^^12^^,^^13^^)^. Although this therapy effectively reduces the microbial load, it does not always result in optimal clinical improvement in all patients, particularly in severe cases. This is primarily because therapy focused solely on bacterial elimination fails to address the host's excessive and destructive inflammatory response ^(^^14^^,^^15^^)^. Therefore, additional strategies are needed that can modulate inflammation and support long-term stability of outcomes, especially in patients who are more susceptible to chronic inflammatory responses and less responsive to conventional therapies.

Host modulation therapy (HMT) is an approach that re-regulates the host immune response to suppress excessive inflammation by balancing anti- and pro-inflammatory mediators, thereby preventing and repairing periodontal tissue damage ^(^^16^^,^^17^^)^. In periodontitis treatment, HMT is used to complement the effectiveness of NSPT which is often limited in patients with chronic inflammatory responses, so that clinical results can be more stable and optimal ^18^. Several agents, including aspirin and nonsteroidal anti-inflammatory drugs (NSAID), have been used in this HMT approach, but their long-term use is limited by the adverse effect risks, including gastrointestinal mucosal damage, renal toxicity, and drug hypersensitivity ^(^^16^^,^^19^^-^^21^^)^. Therefore, there is a need for HMT agents that have a better safety profile and provide consistent clinical effects, for which natural agents with anti-inflammatory properties are an attractive option to be evaluated in the context of adjunct therapy for periodontitis ^22^^-^^24^.

Omega-3 fatty acids (n3-FA), a class of polyunsaturated fatty acids (PUFA), are essential for human health; although they cannot be synthesized endogenously, but can be found abundantly in nature ^(^^25^^,^^26^^)^. Some n3-FA components that have been widely researched and provide health benefits include α-linoleic acid (ALA), typically derived from various nuts and seeds, as well as eicosapentaenoic acid (EPA) and docosahexaenoic acid (DHA), both of which are primarily obtained from fish oil ^27^. Evidence has found beneficial effects of n3-FA on health, including a lower risk of various chronic systemic diseases ^28^. Among these benefits are their anti-inflammatory and immunomodulatory properties, which are particularly relevant to periodontitis management. This is based on the n3-FA mechanism through modulation of immune balance, oxidative stress, and inflammation, thus providing protective and therapeutic effects in periodontitis ^(^^29^^,^^30^^)^.

Although numerous clinical trials evaluating adjunctive n3-FA for SRP have been conducted over the past decade; the results have been inconsistent, with some reporting significant benefits, while others showed no significant improvement. In addition, several meta-analyses have also been conducted, but one previous meta-analysis only evaluated the effects over a three-month period ^31^, three meta-analyses combined RCTs with additional interventions for n3-FA and SRP in the form of aspirin, which may affect the results when compared with n3-FA and SRP alone ^31^^-^^33^, and three meta-analyses involved patients with diabetes mellitus ^31^^,^^32^^,^^34^, which may affect the results of the studies because periodontitis and diabetes mellitus have a bidirectional relationship ^35^. Therefore, this meta-analysis aimed to systematically synthesize evidence from RCTs investigating the n3-FA supplementation as adjunct to SRP in periodontitis, as well as update the findings of previous meta-analyses by including recent RCTs.

## METHODS

### Registration

This systematic review and meta-analysis was conducted following the PRISMA 2020 reporting guidelines ^36^. Registration for the protocol of this study has been done at PROSPERO with registration number CRD420251248258.

### Eligibility criteria

The focused question in this study was formulated using the Population, Intervention, Comparison, Outcomes, and Study design (PICOS) framework. Accordingly, the research question was “In adult patients with clinically diagnosed periodontitis (P), does n3-FA supplementation administered after SRP (I) compared to SRP with or without placebo (C) lead to improvements in probing pocket depth (PPD) and clinical attachment level (CAL) (O) in RCTs with a parallel-group design (S)?”.

According to the PICOS model formulated, studies were retrieved if: 1) RCTs with at least two study groups consisting of a test group (n3-FA supplementation administered after SRP) and a control group (SRP with or without additional placebo); 2) trials administering n3-FA or fish oil capsules with a clear total dose or EPA and DHA dose details; 3) studies involving adult patients (≥18 years) with moderate to severe periodontitis or stage II to IV periodontitis, defined either according to the 2017 American Academy of Periodontology (AAP) case definition or according to pre-2017 diagnostic criteria ^37^^-^^39^; 4) studies reporting baseline and final values of PPD and/or CAL outcomes, expressed as means and standard deviations (SD); 5) RCTs with n3-FA administration and a follow-up duration of at least 3 months after intervention; and 6) full-text, peer-reviewed articles, with no restriction on publication year or language.

Studies were excluded if: 1) the intervention study used a non-randomized design; 2) studies involving patients with gingivitis, mild periodontitis, stage I periodontitis, or periodontitis characterized by PPD ≤4 mm or CAL ≤2 mm ^37^; 3) RCTs involved combination interventions such as n3-FA plus aspirin, n3-FA plus standard adjunctive therapy, or any combination of agents that could influence the effect of n3-FA supplementation; 4) the study provided or involved surgical periodontal treatment; 5) RCTs reported outcomes not in numerical format, such as data presented only in graphs or diagrams; 6) the study had incomplete data, including missing baseline or end-of-intervention data; and 7) the study was a review, case study, animal study, or in vitro study.

### Information sources

A comprehensive and systematic search was implemented to identify RCTs evaluating the effects of n3-FA supplementation as adjunct to SRP in periodontitis. Databases used included Scopus, PubMed, Web of Science, Cochrane Library, and EBSCO. In addition, backward citation searching against the bibliography of included studies and forward citation searching via Google Scholar were carried out to ensure that all relevant publications were identified.

### Search strategy

In the literature search, search strategies were constructed using a combination of MeSH, free-text terms, and Boolean operators, adapted to the characteristics of each database. Basic keywords for the intervention included “omega-3 fatty acids” OR “polyunsaturated fatty acids” OR “eicosapentaenoic acid” OR “docosahexaenoic acid” OR “fish oil”. These terms were subsequently combined with periodontal-related keywords using the Boolean operator “AND”, namely “periodontitis” OR “periodontal disease” OR “dental scaling” OR “root planing”. Details of queries in each database were presented in Table 1. This search was performed by two investigators (A.I. and F.M.R.) in August 2025.

**Table 1:** Detailed queries in each database

Database	Queries
Scopus	TITLE-ABS-KEY ( “omega-3 fatty acids” OR “polyunsaturated fatty acids” OR “eicosapentaenoic acid” OR “docosahexaenoic acid” OR “fish oil” ) AND TITLE-ABS-KEY ( periodontitis OR “periodontal disease” OR “dental scaling” OR “root planing” ) AND NOT TITLE-ABS-KEY ("review" OR "narrative review" OR "literature review" OR "scoping review" OR "rapid review" OR "umbrella review" OR "integrative review" OR "critical review" OR "systematic review" OR "systematic review and meta-analysis" OR "meta-analysis" OR "case study" OR "case report" OR "case series" OR "commentary" OR "editorial" OR "short communication" OR "animal study" OR "animal" OR "in vivo" OR "mouse" OR "mice" OR "murine" )
PubMed	#1 ("fatty acids omega 3"[Supplementary Concept] OR "fatty acids omega 3"[All Fields] OR "omega 3 fatty acids"[All Fields] OR "fatty acids, omega 3"[MeSH Terms] OR ("fatty"[All Fields] AND "acids"[All Fields] AND "omega 3"[All Fields]) OR "omega 3 fatty acids"[All Fields] OR ("fatty acids unsaturated"[Supplementary Concept] OR "fatty acids unsaturated"[All Fields] OR "polyunsaturated fatty acids"[All Fields] OR "fatty acids, unsaturated"[MeSH Terms] OR ("fatty"[All Fields] AND "acids"[All Fields] AND "unsaturated"[All Fields]) OR "unsaturated fatty acids"[All Fields] OR ("polyunsaturated"[All Fields] AND "fatty"[All Fields] AND "acids"[All Fields]) #2 ("eicosapentaenoic acid"[Supplementary Concept] OR "eicosapentaenoic acid"[All Fields] OR "eicosapentaenoic acid"[MeSH Terms] OR ("eicosapentaenoic"[All Fields] AND "acid"[All Fields]) #3 ("docosahexaenoic acids"[Supplementary Concept] OR "docosahexaenoic acids"[All Fields] OR "docosahexaenoic acid"[All Fields] OR "docosahexaenoic acids"[MeSH Terms] OR ("docosahexaenoic"[All Fields] AND "acids"[All Fields]) OR ("docosahexaenoic"[All Fields] AND "acid"[All Fields]) #4 ("fish oils"[Supplementary Concept] OR "fish oils"[All Fields] OR "fish oil"[All Fields] OR "fish oils"[MeSH Terms] OR ("fish"[All Fields] AND "oils"[All Fields]) OR ("fish"[All Fields] AND "oil"[All Fields]) #5 ("periodontal"[All Fields] OR "periodontally"[All Fields] OR "periodontically"[All Fields] OR "periodontics"[MeSH Terms] OR "periodontics"[All Fields] OR "periodontic"[All Fields] OR "periodontitis"[MeSH Terms] OR "periodontitis"[All Fields] OR "periodontitides"[All Fields] OR ("periodontal diseases"[MeSH Terms] OR ("periodontal"[All Fields] AND "diseases"[All Fields]) OR "periodontal diseases"[All Fields] OR ("periodontal"[All Fields] AND "disease"[All Fields]) OR "periodontal disease"[All Fields]) #6 ("dental scaling"[MeSH Terms] OR ("dental"[All Fields] AND "scaling"[All Fields]) OR "dental scaling"[All Fields]) #7 ("root planing"[MeSH Terms] OR ("root"[All Fields] AND "planing"[All Fields]) OR "root planing"[All Fields]) #8 ("review" OR "narrative review" OR "literature review" OR "scoping review" OR "rapid review" OR "umbrella review" OR "integrative review" OR "critical review" OR "systematic review" OR "systematic review and meta-analysis" OR "meta-analysis" OR "case study" OR "case report" OR "case series" OR "commentary" OR "editorial" OR "short communication" OR "animal study" OR "animal" OR "in vivo" OR "mouse" OR "mice" OR "murine") #9 (#1 OR #2 OR #3 OR #4) AND (#5 OR #6 OR #7) NOT #8
Web of Science	#1 “omega-3 fatty acids” OR “polyunsaturated fatty acids” OR “eicosapentaenoic acid” OR “docosahexaenoic acid” OR “fish oil” #2 periodontitis OR “periodontal disease” OR “dental scaling” OR “root planing” #3 review OR "narrative review" OR "literature review" OR "scoping review" OR "rapid review" OR "umbrella review" OR "integrative review" OR "critical review" OR "systematic review" OR "systematic review and meta-analysis" OR meta-analysis OR "case study" OR "case report" OR "case series" OR commentary OR editorial OR "short communication" OR "animal study" OR animal OR "in vivo" OR mouse OR mice OR murine #4 #1 (Topic) AND #2 (Topic) NOT #3 (Title)
Cochrane Library	Title Abstract Keyword: (“omega-3 fatty acids” OR “polyunsaturated fatty acids” OR “eicosapentaenoic acid” OR “docosahexaenoic acid” OR “fish oil”) AND (periodontitis OR “periodontal disease” OR “dental scaling” OR “root planing”)
EBSCO	(TI(“omega-3 fatty acids” OR “polyunsaturated fatty acids” OR “eicosapentaenoic acid” OR “docosahexaenoic acid” OR “fish oil”) OR AB(“omega-3 fatty acids” OR “polyunsaturated fatty acids” OR “eicosapentaenoic acid” OR “docosahexaenoic acid” OR “fish oil”) OR SU(“omega-3 fatty acids” OR “polyunsaturated fatty acids” OR “eicosapentaenoic acid” OR “docosahexaenoic acid” OR “fish oil”)) AND (TI(periodontitis OR “periodontal disease” OR “dental scaling” OR “root planing”) OR AB(periodontitis OR “periodontal disease” OR “dental scaling” OR “root planing”) OR SU(periodontitis OR “periodontal disease” OR “dental scaling” OR “root planing”)) NOT (TI("review" OR "narrative review" OR "literature review" OR "scoping review" OR "rapid review" OR "umbrella review" OR "integrative review" OR "critical review" OR "systematic review" OR "systematic review and meta-analysis" OR "meta-analysis" OR "case study" OR "case report" OR "case series" OR "commentary" OR "editorial" OR "short communication" OR "animal study" OR "animal" OR "in vivo" OR "mouse" OR "mice" OR "murine"))

### Selection process

All search results were imported into Zotero software version 8.0 for Windows, and deduplication was performed automatically followed by manual verification. Articles were then screened at an initial stage to eliminate irrelevant publications. Publications that were deemed potentially eligible were then obtained in full text for detailed evaluation. The PICOS model along with pre-defined inclusion and exclusion criteria was applied in this stage to determine final eligibility. RCTs that satisfied the eligibility criteria in this final stage were included for analysis, both qualitatively and quantitatively; while the specific reasons for excluding studies were documented and reported. This selection process was done by two researchers (A.I. and F.M.R.). Discrepancies that emerged throughout the process were addressed through deliberation until a unanimous decision was achieved. If decision could not be achieved, final decision was adjudicated by the third author (R.L.). This entire selection process was illustrated using a PRISMA flowchart.

### Data collection process

This process was conducted by two authors (F.M.R. and R.A.) by reading the full-text and extracting important data using Microsoft Excel software version 2016 for Windows. Important information extracted included study/authors, country, study design, participants, age, diagnostic criteria for periodontitis or periodontal status at baseline, study duration, intervention details (type/form and daily dose of n3-FA), EPA and DHA dose, supplementation duration, controls, and information related to adverse events. Data that were not available, such as information regarding adverse events, or could not be extracted from the study characteristics, were recorded as “not available (NA).”

For primary clinical outcomes, numerical data from each study were extracted for meta-analysis purposes, including the final number of participants analyzed in both groups, as well as mean values and SDs for PPD and CAL measured at baseline and at the completion of the intervention. This analysis used final values, and to ensure the appropriateness of using final values, baseline balance between groups was verified by comparing means and SD of PPD and CAL at baseline between groups using random-effects meta-analytic approach. The secondary outcome included adverse events reported by included studies.

### Risk of bias assessment

Risk of bias (RoB) was evaluated using the Cochrane RoB2 tool ^40^. This assessment was performed following the assessment guidelines provided by Cochrane RoB2, including evaluation of bias related to the randomization process, deviation from the intended intervention, missing outcome data, outcome measurement, and selection of the reported result. Each domain was rated as presenting a low, some concerns, or high RoB. Overall RoB was determined based on the overall results of the assessment for each domain. The outcomes of RoB were summarized and graphically depicted using the RobVis tool ^41^. This RoB assessment was independently conducted by two reviewers (F.M.R. and R.L.), who had previously calibrated their interpretation of the signaling questions and domain-specific criteria. Disagreements encountered during the evaluation were addressed through thorough deliberation to achieve consensus, with input from a senior researcher (A.I.).

### Statistical analysis

Statistical analyses were performed using PPD and CAL outcomes reported as means and SDs. If any included studies had multiple intervention arms, only the n3-FA supplementation and control arms were selected for inclusion in meta-analysis. Non-relevant intervention arms were excluded to maintain independent comparisons. Random-effects model was applied for the meta-analyses, given the expected heterogeneity across studies arising from variations in periodontitis criteria, baseline periodontal severity, n3-FA dosage, and duration of supplementation. Meta-analysis results were then reported as mean differences (MD) and 95% confidence intervals (CI). An effect was considered statistically significant when the 95% CI that did not cross zero and *p*-value < 0.05.

Heterogeneity was evaluated using the χ^2^ test, with statistical significance defined as *p* <0.1. The extent of heterogeneity was quantified using the *I* ^*2*^ statistic, where values <25%, 25-75%, and >75% were interpreted as reflecting low, moderate, and high heterogeneity, respectively ^42^. Subgroup analyses stratified by supplementation duration (3 and 6 months) and EPA + DHA daily dose (<1,000 and ≥1,000 mg/day) were performed. The 1,000 mg/day cut-off was selected based on the dose distribution across the included studies. Additionally, subgroup analysis based on the risk of bias was also conducted. A leave-one-out sensitivity analysis was performed to assess the robustness of the pooled estimates. Publication bias was not analyzed because this meta-analysis included less than 10 publications ^43^. RevMan version 5.4 for Windows was used for statistical analyses, and StataMP version 17 for Windows was utilized to visualize the leave-one-out sensitivity analysis.

### Certainty of evidence

Certainty of evidence was appraised by two authors (A.I. and F.M.R.) using the Grading of Recommendations Assessment, Development and Evaluation (GRADE) ^44^. This approach evaluates evidence quality across the following domains: RoB, inconsistency, indirectness, imprecision, and publication bias. For each outcome, the final certainty rating reflects the level of confidence that the observed effect estimate represents the true effect. Four levels of evidence were applied: very low, low, moderate, and high.

## RESULTS

### Study selection

Database search identified 1,023 records. After 131 duplicates were removed, 892 records proceeded for initial screening based on title and abstract relevance. Due to irrelevance, 815 records were not retrieved, leaving 77 reports for full-text assessment. Sixty-nine publications were excluded for several reasons, including being review studies, animal studies, non-randomized designs, and case reports. Additional exclusions were applied to studies that did not conform to the predetermined PICOS criteria. Consequently, eight RCTs met the eligibility criteria from the electronic database search.

**Figure 1:**
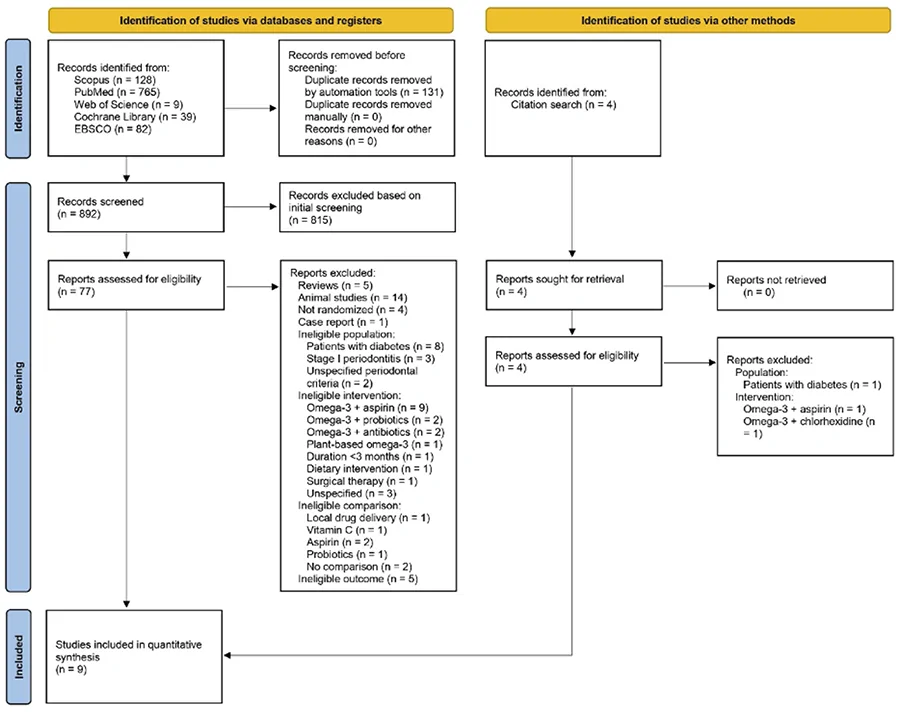
PRISMA flowchart

In addition, the citation search yielded four records, of which three were excluded because they were irrelevant to the population and intervention. Therefore, one study was obtained from the citation search. Finally, 9 RCTs ^45^^-^^53^ were finally included for qualitative and quantitative synthesis. The selection process was systematically reported in the PRISMA flowchart (Figure 1).

### Study characteristics

Nine RCTs evaluating n3-FA supplementation as an adjunct therapy for SRP were included. All participants were adult patients with clinically diagnosed moderate-advanced or stage II-IV chronic periodontitis. Five studies used pre-2017 periodontitis criteria, including moderate to severe chronic periodontitis ^45^^,^^49^, generalized chronic periodontitis ^46^^,^^53^, and advanced chronic periodontitis ^47^. Four studies used the 2017 AAP diagnostic criteria, including one study ^50^ involved patients with stage II to III grade B periodontitis, one study ^48^ involved patients with stage II to IV grade B periodontitis, and two others ^51^^,^^52^ involved patients diagnosed with stage III and IV periodontitis. Geographically, the studies were conducted in several regions, including India ^45^^,^^53^, Egypt ^46^^,^^47^^,^^50^, Iran ^48^, Iraq ^49^, and Poland ^51^^,^^52^. Across all included RCTs, participants in both the treatment and control arms underwent SRP and received oral hygiene instructions (Table 2).

**Table 2:** General study characteristics

Study	Country	Study design	Participants (intervention / control)	Age range (mean, intervention / control)	Periodontal status (baseline)	Study duration
Deore et al. ^45^	India	Parallel, double-blind, RCT	60 (30/30)	30-60 (45.40 ± 40.90/44.47 ± 5.20)	Moderate chronic periodontitis; PPD ≥5 mm; CAL ≥4 mm Severe chronic periodontitis; PPD ≥5 mm; CAL ≥6 mm	3 months
Elgendy et al. ^46^	Egypt	Parallel, double-blind, RCT	50 (25/25)	45-60 (50.24 ± 3.05/51.44 ± 3.36)	Generalized chronic periodontitis; PPD ≥5 mm	6 months
El-Sharkawy et al. ^47^	Egypt	RCT	34 (17/17)	35-60 (45.75 ± 2.05/47.82 ± 2.21)	Advanced chronic periodontitis; PPD >6 mm; CAL ≥4 mm; RBL evidence	3 months
Maybodi et al. ^48^	Iran	Parallel, double-blind, RCT	30 (15/15)	30-70 (45.467 ± 7.698/42.867 ± 8.416)	Stage II-IV grade B periodontitis; CAL ≥3 mm	3 months
Salman et al. ^(49^)	Iraq	Single-blind, RCT	50 (25/25)	30-60 (NA/NA)	Chronic periodontitis; PPD ≥5 mm	3 months
Shalaby et al. ^50^	Egypt	RCT	30 (15/15)	35-55 (NA/NA)	Stage II-III grade B periodontitis; CAL ≤5 mm (stage II) or ≥5 mm (stage III); RBL >30% (stage II) or extending to the mid-third of the root length (stage III)	6 months
Stańdo et al. ^51^	Poland	Parallel-arm, RCT	40 (20/20)	22-70 (47.5 ± 9.63/49.3 ± 12.80)	Generalized stage III-IV periodontitis; CAL ≥5 mm; PPD ≥6 mm; RBL >1/3 of the root length	3 months
Stańdo-Retecka et al. ^52^	Poland	Parallel-arm, RCT	40 (20/20)	22-70 (44.1 ± 7.7/52.2 ± 10.8)	Stage III/IV periodontitis; PPD ≥6 mm; CAL ≥5 mm; RBL extending 1/3 of the root length	6 months
Umrania et al. ^53^	India	Examiner-masked, RCT	40 (20/20)	NA (44 ± 6.44/43.5 ± 5.8)	Chronic generalized periodontitis; CAL ≥5 mm	3 months

RCT, randomized controlled trials; PPD, probing pocket depth; CAL, clinical attachment level; RBL, radiographic bone loss; NA, not available.

According to the intervention, all RCTs reported the specific composition of n3-FA, where three studies ^(^^45^^,^^48^^,^^53^^)^ used a total dose of 300 mg of EPA plus DHA per day, one study ^47^ administered 600 mg/day, another study ^50^ administered 660 mg/day, two studies ^46^^,^^49^ used 1,000 mg/day, and two studies ^(^^51^^,^^52^^)^ used 4,400 mg/day. Finally, the duration of n3-FA supplementation and the follow-up period varied among studies, ranging from 3 months ^(^^45^^,^^47^^-^^49^^,^^51^^,^^53^^)^ to 6 months ^(^^46^^,^^50^^,^^52^^)^ (Table 3).

**Table 3:** Intervention and control details of included studies

Study	Intervention	EPA + DHA dose	Control	Adverse events
Deore et al. ^45^	SRP; 300 mg/day n3-FA (180 mg EPA/120 mg DHA) for 3 months; oral hygiene instructions	300 mg/day	SRP; placebo capsules; oral hygiene instructions	NA
Elgendy et al. ^46^	SRP; 1000 mg n3-FA capsules (300 mg EPA/200 mg DHA), twice/day for 6 months; oral hygiene instructions	1,000 mg/day	SRP; placebo (olive oil) capsules twice a day; oral hygiene instructions	No adverse side effects
El-Sharkawy et al. ^47^	SRP; 2000 mg/day fish oil capsules containing n3-FA (30% EPA/DHA) for 3 months; oral hygiene instructions	600 mg/day	SRP; oral hygiene instructions	No gastrointestinal disturbances or other adverse events
Maybodi et al. ^48^	SRP; 1000 mg/day n3-FA soft-gels (300 mg n3-FA marine triglycerides/180 mg EPA/120 mg DHA) for 3 months; oral hygiene instructions	300 mg/day	SRP; placebo (soft-gels containing 150 mg soybean oil); oral hygiene instructions	No adverse side effects
Salman et al. ^49^	SRP; 1000 mg/day EPA and DHA n3-FA for 3 months; oral hygiene instructions	1,000 mg/day	SRP; placebo; oral hygiene instructions	NA
Shalaby et al. ^50^	SRP; 3000 mg/day n3-FA (13% EPA/9% DHA) for 6 months; oral hygiene instructions	660 mg/day	SRP; oral hygiene instructions	NA
Stańdo et al. ^51^	SRP; 20 mL/day n3-FA (2.6 g EPA/1.8 g DHA) for 3 months; oral hygiene instructions	4,400 mg/day	SRP; oral hygiene instructions	Six participants reported nausea and a disturbing fishy-smelling halitosis
Stańdo-Retecka et al. ^52^	SRP; 20 mL/day fish oil containing n3-FA (2.6 g EPA/1.8 g DHA) for 6 months; oral hygiene instructions	4,400 mg/day	SRP; oral hygiene instructions	Eight patients reported nausea and a disturbing fishy-smelling halitosis
Umrania et al. ^53^	SRP; 700 mg/day n3-FA capsules (180 mg EPA/120 mg DHA) for 3 months; oral hygiene instructions	300 mg/day	SRP; oral hygiene instructions	NA

n3-FA, omega-3 fatty acids; EPA, eicosapentaenoic acid; DHA, docosahexaenoic acid; SRP, scaling and root planing; NA, not available.

### Risk of bias in studies

**Figure 2:**
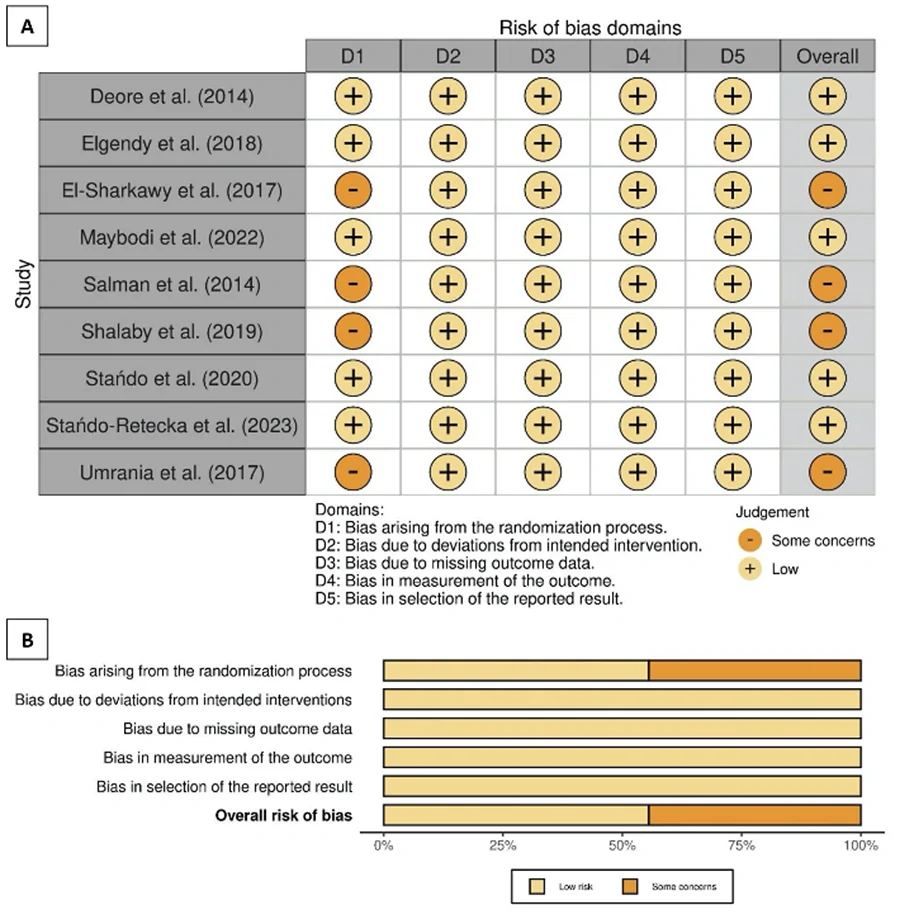
Risk of bias assessment using Cochrane RoB2 tool: Summary (A) and graph (B).

Five RCTs were judged as presenting low risk ^(^^45^^,^^46^^,^^48^^,^^51^^,^^52^^)^. Meanwhile, four others ^(^^47^^,^^49^^,^^50^^,^^53^^)^ were judged as presenting some concerns. No domain was judged as having high risk (Figure 2).

### Meta-analysis for probing pocket depth

**Figure 3:**
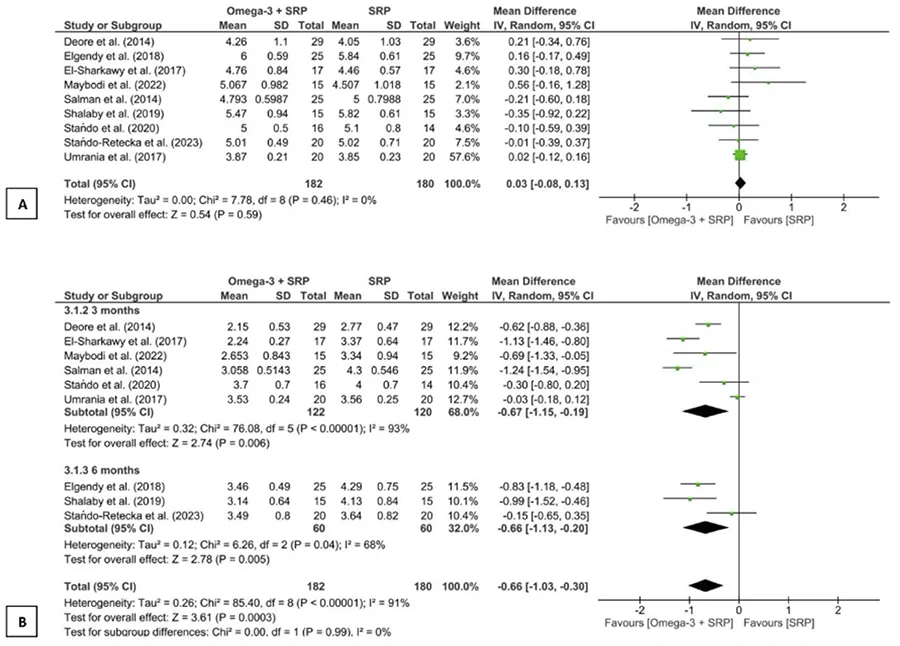
Forest plot: (A) Baseline PPD values and (B) efficacy of n3-FA supplementation on PPD.

Nine RCTs involving 362 participants (182 in intervention/180 in control) were included in meta-analysis evaluating n3-FA supplementation on PPD. Baseline PPD values demonstrated no meaningful differences between groups (MD = 0.03, 95% CI: -0.08, 0.13; *p* = 0.59), with no heterogeneity observed (*p* = 0.46; *I* ^*2*^ = 0%) (Figure 3A).

Pooled analysis demonstrated that adjunctive n3-FA supplementation following SRP resulted in a 0.66 mm greater reduction in PPD compared to SRP alone (MD = -0.66; 95% CI: -1.03, -0.30; *p* = 0.0003). However, substantial heterogeneity across studies was observed (*p* <0.00001; *I* ^*2*^ = 91%) (Figure 3B).

Subgroup analysis stratified by duration of supplementation at 3 and 6 months, the PPD reduction was 0.67 mm (MD = -0.67; 95% CI: -1.15, -0.19; p = 0.006) and 0.66 mm (MD = -0.66; 95% CI: -1.13, -0.20; *p* = 0.005), respectively, which was greater than SRP alone. Heterogeneity remained high in studies with duration of 3 months (*p* <0.00001; *I* ^*2*^ = 93%), but decreased to moderate heterogeneity in studies with duration of 6 months (*p* = 0.04; *I* ^*2*^ = 68%) (Figure 3B).

Subgroup meta-analysis was also performed based on n3-FA dosage. All studies specifically reported EPA and DHA dosages, with the dose range being 300-4,400 mg/day. Meta-analysis of five studies ^(^^45^^,^^47^^,^^48^^,^^50^^,^^53^^)^ showed that EPA + DHA doses of <1,000 mg/day resulted in a 0.67-mm reduction in PPD (MD = -0.67; 95% CI: -1.15, -0.06; p = 0.007; I^2^ = 92%), and four studies ^(^^46^^,^^49^^,^^51^^,^^52^^)^ with doses of ≥1,000 mg/day resulted in a 0.66-mm reduction (MD = -0.66; 95% CI: -1.16, -0.16; p = 0.009; I^2^ = 84%) compared to SRP alone (Data not shown).

Stratified by study quality, five studies ^(^^45^^,^^46^^,^^48^^,^^51^^,^^52^^)^ assessed as low RoB resulted in a PPD reduction of 0.56 mm with a decrease in heterogeneity to moderate (MD = -0.56; 95% CI: -0.79; -0.33; p <0.00001; I^2^ = 35%). Meanwhile, four studies ^(^^47^^,^^49^^,^^50^^,^^53^^)^ with moderate RoB resulted in a PPD reduction of 0.84 mm with increased heterogeneity (MD = -0.84; 95% CI: -1.57; -0.10; p = 0.03; I^2^ = 96%) (Data not shown).

### Meta-analysis for clinical attachment level

**Figure 4:**
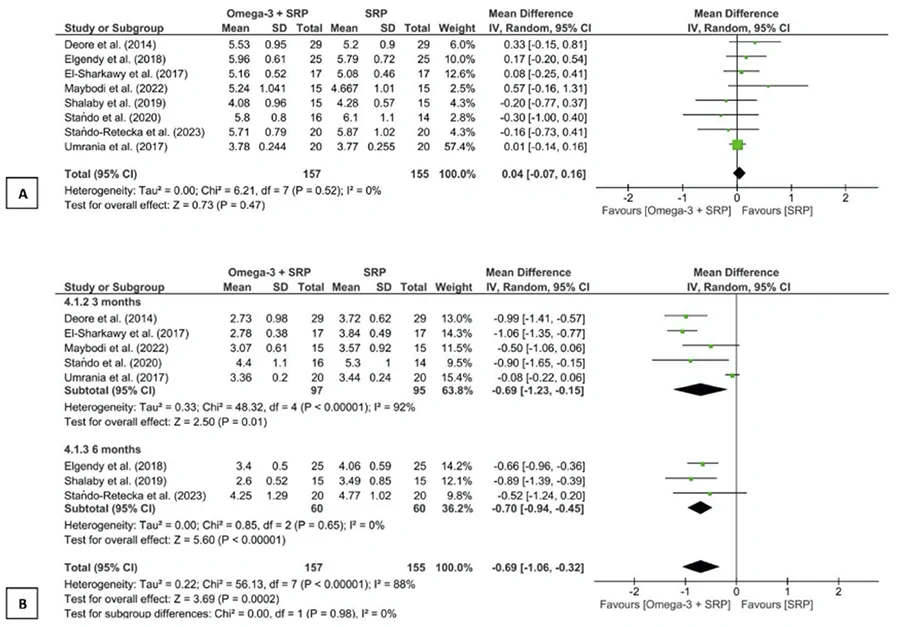
Forest plot: (A) Baseline CAL values and (B) efficacy of n3-FA supplementation on CAL.

Eight RCTs involving 312 participants (157 in intervention/155 in control) were analyzed for CAL outcome. Baseline CAL values between groups demonstrated no noteworthy differences (MD = 0.04, 95% CI: -0.07, 0.16; p = 0.47), with no evidence of heterogeneity (p = 0.52; I^2^ = 0%) (Figure 4A).

Pooled analysis showed that adjunctive n3-FA supplementation following SRP resulted in a 0.69 mm greater gain in CAL compared to SRP alone (MD = -0.69; 95% CI: -1.06, -0.32; p = 0.0002). Nonetheless, substantial heterogeneity across studies was observed (p <0.00001; I^2^ = 88%) (Figure 4B).

Subgroup analysis stratified by duration of intervention at 3 and 6 months, CAL increased by 0.69 mm (MD = -0.69; 95% CI: -1.23, -0.15; *p* = 0.01) and 0.70 mm (MD = -0.70; 95% CI: -0.94, -0.45; p <0.00001), respectively. Substantial heterogeneity was observed in studies administering n3-FA for 3 months (p <0.00001; I^2^ = 92%), but was not observed in studies administering for 6 months (p = 0.65; I^2^ = 0%) (Figure 4B).

Subgroup analysis was also performed stratified by n3-FA doses, with the EPA plus DHA dose range being 300-4,400 mg/day. Meta-analysis included five studies ^(^^45^^,^^47^^,^^48^^,^^50^^,^^53^^)^ showed that EPA + DHA doses of <1,000 mg/day resulted in a 0.69 mm increase in CAL (MD = -0.69; 95% CI: -1.22, -0.17; p = 0.009; I^2^ = 92%), and three studies ^(^^46^^,^^51^^,^^52^^)^ with doses of ≥1,000 mg/day resulted in a 0.67 mm (MD = -0.67; 95% CI: -0.93, -0.41; p <0.00001; I^2^ = 0%) compared to SRP (Data not shown).

Based on the RoB, five studies ^(^^45^^,^^46^^,^^48^^,^^51^^,^^52^^)^ with low RoB resulted in a CAL gain of 0.72 mm with a decrease in heterogeneity to low (MD = -0.72; 95% CI: -0.93; -0.52; p < 0.00001; I^2^ = 0%). While three studies ^(^^47^^,^^50^^,^^53^^)^ with moderate RoB produced a non-significant CAL gain of 0.66 mm with substantial heterogeneity (MD = -0.66; 95% CI: -1.40; 0.08; p = 0.08; I^2^ = 95%) (Data not shown).

### Sensitivity analysis

**Figure 5:**
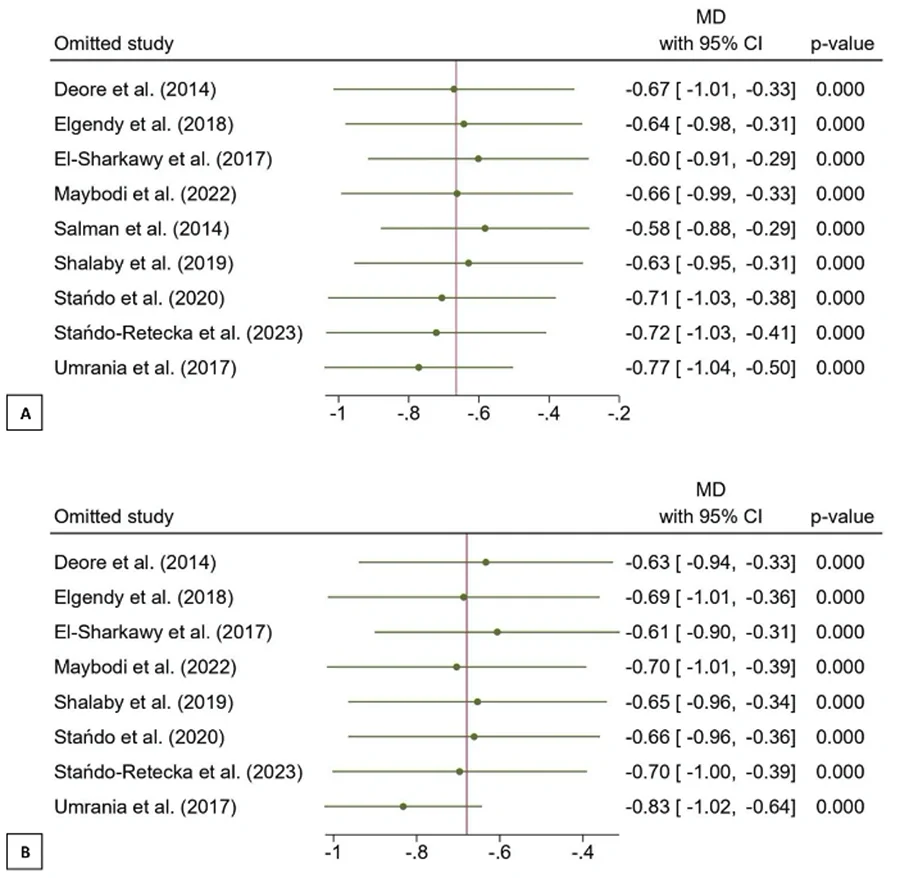
Leave-one-out sensitivity analysis: (A) PPD outcome and (B) CAL outcome.

The leave-one-out sensitivity analysis showed that the meta-analyses for PPD (Figure 5A) and CAL outcomes (Table 5B) were both stable.

### Adverse events

Three of nine trials reported no adverse events or side effects in participants receiving n3-FA ^46^^-^^48^. However, 37.5% (6/16) of intervention patients in Stańdo et al. ^51^ and 40% (8/20) patients in Stańdo-Retecka et al. ^52^ reported nausea and bothersome fish-smelling halitosis. It is noteworthy that these side effects occurred in the two trials administering the highest doses of EPA and DHA (4,400 mg/day). In addition, no other adverse side effects were reported. Four studies ^(^^45^^,^^49^^,^^50^^,^^53^^)^ did not provide information regarding adverse events.

### Certainty of evidence

The certainty of evidence regarding the PPD outcome was classified as low. The initial confidence level was rated as high because the included studies were RCTs. However, the certainty was downgraded due to the substantial heterogeneity. Meanwhile, the CAL outcome was also rated as low certainty. The initial confidence similarly started at high due to the inclusion of RCTs, but was downgraded because of the high heterogeneity. Therefore, although pooled estimates indicate favorable effects of n3-FA supplementation on both PPD and CAL, the low certainty of evidence indicates that the true magnitude of the effect may differ from the observed estimates (Table 4).

**Table 4:** Summary of findings

Outcome	Risk of bias	Inconsistency	Imprecision	Indirectness	Publication bias	Certainty
PPD (n = 9 RCTs)	No serious	Very serious	No serious	No serious	Not assessed	Low
CAL (n = 8 RTCs)	No serious	Very serious	No serious	No serious	Not assessed	Low

RCT, randomized controlled trial; PPD, probing pocket depth; CAL, clinical attachment level.

## DISCUSSION

This meta-analysis demonstrated that n3-FA supplementation as adjunct therapy to NSPT was associated with greater clinical improvements, resulting in reductions in PPD and gains in CAL compared to SRP alone. Subgroup analysis further indicated that these favorable effects were consistently observed at both 3- and 6-month durations of supplementation. Additionally, subgroup analysis stratified by EPA plus DHA dose also did not reveal a clear dose-response relationship, as similar clinical improvements were observed with both lower (<1,000 mg/day) and higher (≥1,000 mg/day) doses. Importantly, when stratified by study quality, the magnitude of effect remained significant but became more consistent among studies assessed as having a low RoB, accompanied by a substantial reduction in heterogeneity, particularly for CAL outcome. In contrast, studies with moderate RoB showed larger but more heterogenous and less reliable effect estimates. These findings suggest that the pooled effects observed in the overall analysis are driven primarily by higher-quality evidence and that methodological rigor plays a key role in the stability of estimated treatment effects. Despite the sensitivity analysis demonstrating stable results, the findings should be interpreted cautiously due to the overall substantial heterogeneity and the low level of evidence assigned to both outcomes.

Although the eligibility criteria in this meta-analysis were clearly defined to include only patients with at least moderate or stage II periodontitis, overall substantial heterogeneity remained across the included studies. This residual heterogeneity is likely due to differences in baseline clinical characteristics, including the criteria for defining periodontitis, with some included studies ^(^^45^^-^^47^^,^^49^^,^^53^^)^ using the older 1999 or pre-2017 AAP criteria and others ^(48^^,^^50^^-^^52^^)^ using the more recent or 2017 AAP criteria with staging and/or grading. Even with this restricted spectrum of moderate to severe or stage II to IV periodontitis, variability in baseline periodontal parameters, such as mean PPD, CAL distribution, and case mix, may still influence the magnitude of clinical response to NSPT. Previous evidence suggests that baseline severity of periodontitis is associated with the magnitude of clinical improvement following NSPT ^54^, indicating that residual differences in initial clinical status could contribute to the heterogeneity observed in this systematic review.

In addition, authors believe that the substantial heterogeneity may also be influenced by the variations in n3-FA dosage and duration of supplementation across studies. Interestingly, both high-dose (4,400 mg/day) ^(^^51^^,^^52^^)^ and low-dose (300 mg/day) ^53^ regimens failed to yield significant clinical benefits, irrespective of supplementation duration. These findings suggest that differences in dosage and treatment duration alone may not fully account for the observed heterogeneity, and that other unmeasured factors, such as baseline inflammatory burden, host response variability, or differences in periodontal case mix, may also influence clinical outcomes following adjunctive n3-FA supplementation.

Regarding safety, this systematic review identified two studies ^(^^51^^,^^52^^)^ that reported mild adverse effects, including nausea and fishy halitosis. Notably, these studies administered the highest EPA and DHA n3-FA doses among the included trials (4,400 mg/day). Regulatory guidance from the United States Food and Drug Administration indicates that daily EPA and DHA intake for adults should not exceed 3,000 mg/day, with no more than 2,000 mg/day derived from supplements ^(^^55^^,^^56^^)^. In addition, higher n3-FA intakes have been associated with adverse effects such as gastrointestinal disturbances and, in certain clinical context, increased risk of bleeding and atrial fibrillation ^(^^57^^,^^58^^)^. These findings suggest that while n3-FA supplementation is generally well tolerated, careful consideration of dosage remains important when used as adjunctive therapy, especially at higher dose.

The present findings suggest that n3-FA supplementation may provide additional benefits to periodontal clinical improvement after SRP, reflected by greater reductions in PPD and greater gains in CAL compared to SRP alone. These effects are likely related to the ability of n3-FA to generate specialized pro-resolving mediators, including resolvins, protectins, and maresins, which facilitate resolution of inflammation and support regeneration and repair of periodontal tissue ^59^^-^^62^. n3-FA could thus be viewed as a potential candidate for HMT due to their generally favorable safety profile when used at appropriate doses. Nevertheless, translation of these findings into clinical practice should be approached with caution, given the substantial heterogeneity among studies and the low level of evidence. Thus, n3-FA supplementation may be considered on an individual basis in selected cases, but recommendations for optimal dosage or long-term use cannot yet be established until larger, high-quality RCTs become available.

This meta-analysis has several strengths. First, a comprehensive search strategy across major databases, forward and backward citation tracking, and no restrictions on publication year minimized the likelihood of missing relevant studies. Second, the eligibility criteria were rigorously defined to enhance clinical comparability, restricting inclusion to RCTs involving patients with at least moderate or stage II periodontitis and evaluating n3-FA supplementation as the sole adjunct to SRP without combination with other agents such as aspirin, NSAIDs, antibiotics, or other agents, thereby yielding a more specific effect estimate. Third, subgroup analysis and sensitivity analyses were performed to explore potential sources of heterogeneity and to assess the robustness of the pooled estimates. By incorporating the most recent RCTs, excluding studies with co-interventions such as aspirin or other HMT agents, and restricting the analysis to non-diabetic populations, the present meta-analysis provides a more focused and specific effect estimate than previous meta-analyses ^31^^-^^34^.

Despite these strengths, several limitations should be acknowledged. First, substantial heterogeneity remained for both PPD and CAL outcomes, likely reflecting residual clinical and methodological variability across studies, including differences in baseline periodontal characteristics, diagnostic criteria, n3-FA formulations and dosages, and supplementation duration. Second, the variation in follow-up duration (3 and 6 months) and the lack of adverse event reporting in several studies limit a comprehensive evaluation of long-term efficacy and overall safety. Consequently, the overall certainty of evidence was rated as low, underscoring the need for cautious interpretation of the findings.

Therefore, future research should focus on RCTs with more rigorous methodological designs to reduce heterogeneity and enhance the certainty of evidence regarding the efficacy of n3-FA supplementation as adjunct therapy for periodontitis. Future studies should employ n3-FA formulations with consistent dosing regimens and dose ranges that allow for a more accurate evaluation of potential dose-response relationships. Longer follow-up (6-12 months) are also required to evaluate the durability of clinical effects and to better characterize the long-term safety profile. Furthermore, study populations should be carefully characterized and, where appropriate, stratified according to baseline periodontitis severity or staging and/or grading systems based on the 2017 AAP criteria, to allow clearer interpretation of treatment effects across disease severities. Finally, large, multicenter clinical trials are strongly needed to improve external validity and strengthen the current evidence base.

## CONCLUSIONS

This meta-analysis concludes that n3-FA supplementation as an adjunct therapy to SRP yields greater periodontal clinical improvements than SRP alone, as reflected by significant reductions in PPD and gains in CAL. However, these findings should be interpreted cautiously given the substantial heterogeneity and the low level of evidence. Nevertheless, this study contributes to clarifying the current evidence base and highlights the need for rigorously designed, adequately powered RCTs to confirm the clinical effectiveness and long-term safety of n3-FA as an adjunctive periodontal therapy.
